# Development, diagnosis and therapy of ketosis in non-gravid and non-lactating Guinea pigs

**DOI:** 10.1186/s12917-020-2257-2

**Published:** 2020-02-03

**Authors:** Nicole S. Schmid, Marcus Clauss, Udo Hetzel, Barbara Riond, Monika Bochmann, Jean-Michel Hatt

**Affiliations:** 1 Clinic for Zoo Animals, Exotic Pets and Wildlife, Vetsuisse Faculty, University of Zurich, Winterthurerstrasse 260, CH-8057 Zurich, Switzerland; 2 Institute of Veterinary Pathology, Winterthurerstrasse 268, CH-8057 Zurich, Switzerland; 3 Veterinary Clinical Laboratory, Winterthurerstrasse 260, CH-8057 Zurich, Switzerland; 4 Walter Zoo, CH-9200 Gossau, SG Switzerland

**Keywords:** Guinea pig, Fasting ketosis, Beta-Hydroxybutyrate, Bile acid, Liver damage

## Abstract

**Background:**

Ketosis is a metabolic disorder often triggered by anorexia in animals fed on high energy diets. Although mostly described in pregnant female guinea pigs, under the name of pregnancy toxicosis; there is limited information on ketosis in males and non-pregnant females, often presented to clinics with anorexia or inappetence. The objective of this study was to observe progression of ketosis in guinea pigs, document the changes and evaluate diagnostic methods and a therapeutic approach.

**Results:**

Twenty eight adult guinea pigs (*Cavia porcellus*), castrated males and intact females of obese and slim body condition were fasted for 3 days and refed afterwards. The slim animals served as control group for body condition. Either slim and fat animals were divided into two treatment groups: half of them received fluid replacements with glucose subcutaneously, the other half did not receive any injection and served as treatment control. Serum beta-hydroxybutyrate, and urine acetoacetate and acetone were measured during and after fasting. Serum ALT, bile acids and liver histology were also analyzed after 7 days of refeeding (and therapy). Females and obese guinea pigs showed a significantly higher increase in ketone bodies in serum and urine. Obese, female, or animals not receiving therapy needed more time to regulate ketone bodies to normal levels than slim animals, males or animals receiving therapy. Liver histology revealed increased hepatocyte degeneration and higher glycogen content in obese animals and animals receiving therapy, and additionally more glycogen content in males. Only minor hepatic fat accumulation was documented. Bile acids showed good correlation to histological liver changes whereas ALT did not.

**Conclusions:**

Female and obese animals react more intensively to fasting. As preventive management, animals should be kept in adequate body condition, fasting should be avoided, and anorexia should be treated immediately. In such a case, urinary dip sticks to detect ketone bodies are a useful diagnostic tool. Glucose therapy leads to faster cessation of ketogenesis and should be recommended in cases of ketosis. However, it needs to be adjusted to avoid hepatocyte glycogen overload and degeneration. Measuring bile acids presents a valuable indicator of liver damage.

## Background

The guinea pig (*Cavia porcellus*) is one of the most frequently presented small pets at clinics [1, 2]. Guinea pigs often show unspecific signs of depression and inappetence, mostly as a result of an underlying disease. In case of delayed or absent treatment, there is a risk of secondary or additional metabolic disorders. One important metabolic disorder is ketosis, often encountered in combination with fatty liver disease, caused by anorexia after a period of feeding on a high energy diet [3, 4]. Ketosis, by definition, is the accumulation of acetoacetate, β-hydroxybutyrate (BHB) and acetone in body fluids as a result of increased fat mobilisation for energy production. Due to an excessive beta-oxidation of fatty acids, more acetyl-coenzyme-A is synthetized than can be used for gluconeogenesis; this leads to an accelerated ketogenesis in hepatocytes [5].

Ketosis is described in various species, from domestic ruminants to rabbits, hamsters, guinea pigs and nonhuman primates as well as humans [6, 7]. Multiple studies reported a variety of clinical signs in relation with ketosis when fasting guinea pigs after a period of feeding an energy-dense diet for different amounts of time. In most cases, pregnant guinea pigs were investigated, and a moderate to severe clinical manifestation of ketosis in obese patients could be induced; by contrast, male or non-pregnant guinea pigs remain asymptomatic or present only subclinical changes [4, 6, 8, 9]. Ketosis of pregnant animals is often referred to as ‘pregnancy toxicosis’ [7, 10, 11]. Predisposing factors are obesity, lack of exercise, large fetal loads and primiparity, a change in diet or environment, heat stress, and possibly a genetic predisposition as well [10, 12]. Lachmann, et al. [4] defend that the syndrome of ketosis is triggered primarily by anorexia and is independent of any other factors such as lactation, pregnancy or gender. However, it is still controversial whether males and non-pregnant females are susceptible to ketosis [4, 9].

Bergmann and Sellers [6] fasted pregnant and non-pregnant guinea pigs for 3 days, during which only the pregnant animals developed clinical signs. Another study showed that fasting non-pregnant females and males for 4 days provoked subclinical ketosis, but did not report when pathological changes started [4]. Ganaway and Allen [9] were able to evoke a syndrome in obese virgin guinea pigs indistinguishable from pregnancy toxemia. In a study on the influence of vitamin C deficiency on ketosis in young non-pregnant female guinea pigs, the animals were fasted for 10 days, and already after 1 to 3 days an elevation of ketone bodies in the blood could be measured; however, there was no significant difference between animals with a vitamin C deficient diet and the vitamin C supplemented control group [13].

Apart from anorexia, guinea pigs affected with clinical ketosis show signs like reduced activity, ruffled hair, respiratory distress, body mass loss, depression, lethargy, apathy, somnolence, prostration, convulsions, muscle spasms, paralysis, coma and death; in the case of pregnancy toxicosis, abortion and stillbirth can be documented as well [4, 6, 9, 10, 12, 14].

Ketosis can be diagnosed by blood or urine analysis. Measuring serum BHB is most sensitive and reflects the progression of the clinical ketosis, as for example BHB represents 80% of the total ketone bodies in cattle [15]. Moreover, BHB is less susceptible to deterioration by storage than acetone and acetoacetate [16]. In cats, ketone bodies are detected earlier and in lower concentrations in the blood than in urine [17]. However, there are no published reference values for guinea pigs. In clinical settings, urine is typically analyzed more often than blood, because sampling is easier and less stressful. Commercially available test strips detect acetoacetate and acetone but not BHB in fresh urine and deliver a semiquantitative result, which should be zero in healthy animals [18].

Further clinical laboratory changes in guinea pigs with ketosis are acidosis, hyperkalemia, hypocalcemia, hypoglycemia, hyperlipemia, and severely elevated serum cholesterol, as well as ketonuria, proteinuria, aciduria and a decreased urine pH in fasting animals [4, 8–10, 12, 14]. Sauer [3] found that fasting-induced ketosis is accompanied by a rapid mobilization of fat depots and therefore an increase of total fatty acids in plasma and concentration in the liver.

Post mortem findings in ketosis typically include significantly more severe fatty livers and hepatic lipidosis in animals fasted after a period of high energy feeding compared to animals fed restrictively [4], and potentially fatty changes in liver, kidneys, adrenal glands and lungs [9]. A retrospective study, based on pathology, reported fatty liver in 72% of guinea pigs with an anamnesis of anorexia; additionally, of all the fatty livers seen, 60% were diagnosed in moderately to highly obese guinea pigs [19].

Suggested treatments for ketosis include replacement fluid therapy with dextrose, glucose, calcium and magnesium sulfate along with nutritional support, if necessary by syringe feeding [7, 10, 12, 14]. Bishop [10] also mentions the use of short-acting corticosteroids as helpful in some cases, and recommends monitoring of blood gases, acid/base-ratio, electrolytes, calcium and phosphorus to monitor the progression of the disease. As treatment attempts are often unsuccessful, prevention is considered to be much more important. Factors that should be avoided include obesity, abrupt changes in diet or environment, and other sources of stress. Additionally, in pregnant animals, an increased supplementation of higher-energy feeds 2 weeks before parturition (to avoid a reduction of energy intake due to the restricted intake capacity) and encouragement of exercise can be beneficial [12].

The present study was undertaken to gain more detailed information about the etiology, pathogenesis, onset, trend and treatment of ketosis in non-pregnant guinea pigs. Different diagnostic methods were evaluated. First, the possibility to detect ketone bodies in urine of guinea pigs by commercially available urinary dip sticks (Combur 9©^1^) and its use as an early diagnostic method was assessed. Secondly, a point of care instrument (POC; FreeStyle Precision Neo^2^) was tested for its accuracy in measuring BHB in blood. Ketone bodies in urine and blood were measured to investigate any differences in the onset, progression and trend as well as the putative synchrony to clinical signs. Additionally, we wanted to test whether a difference between slim and obese animals could be confirmed as well as the beneficial effect of a therapy.

## Results

### Behavioral changes

During the time of fasting, the animals were observed performing coprophagy on a regular basis. They took feces directly from the anus, but also collected their feces off the ground (a behavior termed ‘indirect coprophagy’). These observations were not quantified. Only one animal (number 5), a female belonging to the slim group, showed signs of alopecia due to trichophagia, worsening with time spent individually, which improved only after placing her back into the outside enclosures with other group members.

### Body mass

**Fig. 1:**
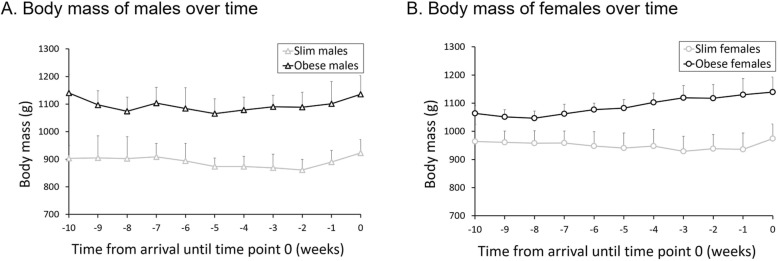
Mean (±SD) of body mass (g) from the day of arrival until the beginning of the study period, of all guinea pigs in one group (*n* = 7, except for slim males *n* = 6). **a** Slim and obese males; **b** Slim and obese females

Slim females (*n* = 7), arriving with a body mass of 963 ± 45 g, did not gain mass during the first observation period (− 3.3 ± 29.5 g; − 0.3 ± 3.0%), whereas obese females (*n* = 7), arriving at 1058 ± 29 g, gained 86.3 ± 61.5 g (8.2 ± 5.9%). In males, body mass gains after castration was similar for slim (start 867 ± 35 g, gain 51.5 ± 35.8 g; 5.9 ± 4.1%; *n* = 6) and obese (start 1071 ± 71 g, gain 45.4 ± 52.6 g; 4.4 ± 5.2%; *n =* 7) individuals. Correspondingly, the GLM indicated no significant differences in percent body mass gain between genders (*F* = 0.460, *P* = 0.504) and only a tendency for a difference between slim and obese animals (*F* = 3.758, *P* = 0.065), but a significant gender X obesity interaction (*F* = 7.838, *P* = 0.010). At the beginning of the adaptation to the individual cages, the average body mass (±SD) for the individual groups was 972 ± 60 g for slim and 1140 ± 53 g for obese females, and 936 ± 60 g for slim and 1135 ± 67 g for obese males (Fig. 1).

**Fig. 2:**
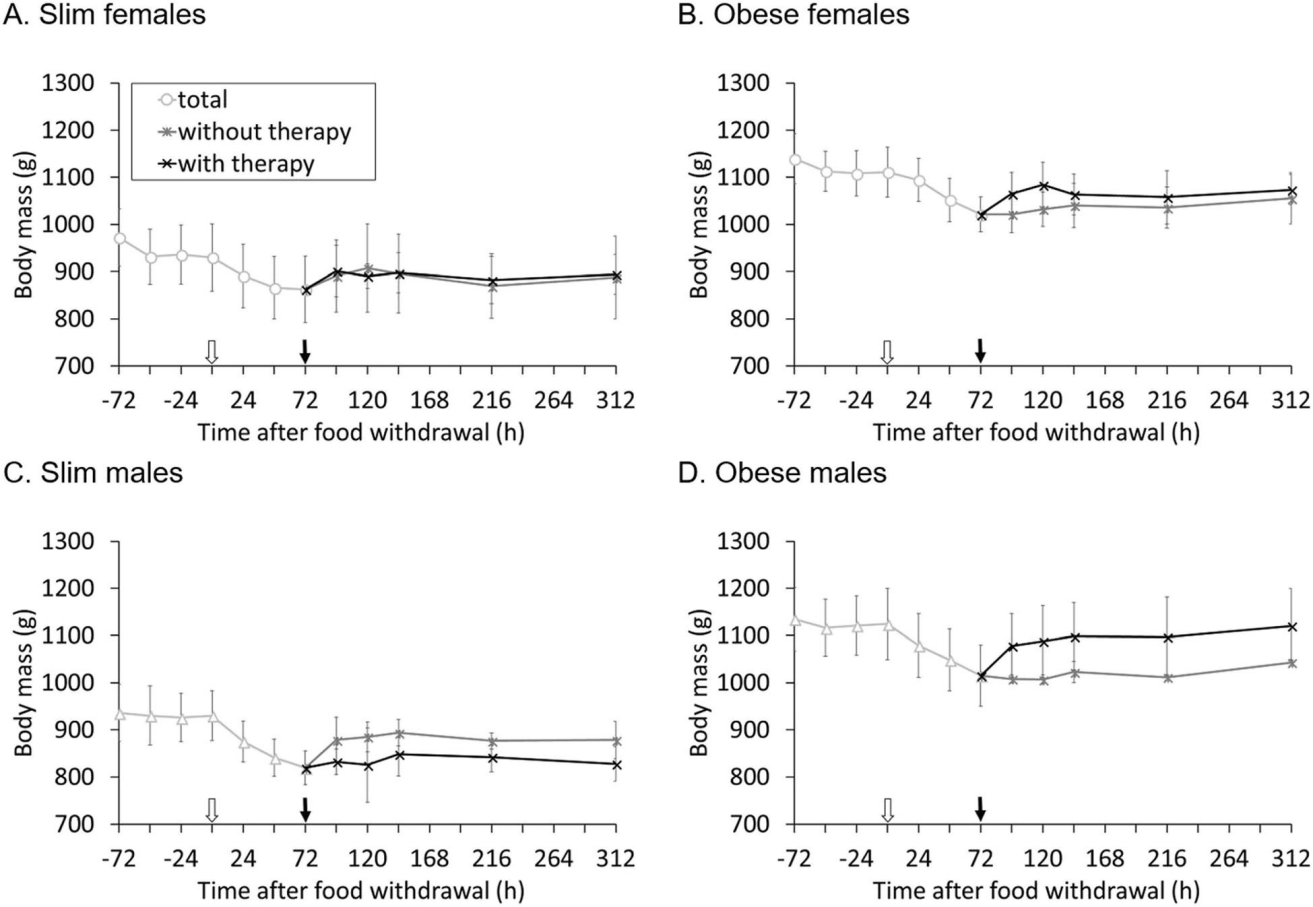
Mean (±SD) body mass (g) of all guinea pigs in one group (*n* = 7, except for slim males *n* = 6); the white and black arrows indicate the beginning and end of the fasting period, respectively. After the end of fasting, animals are divided into therapy groups (*n* = 3/4). **a** Slim females;** b** Obese females; **c** Slim males; **d** Obese males

Fasting the guinea pigs for 3 days caused a body mass loss that differed significantly between groups (*F* = 6.403, *P* = 0.003), with no differences between slim and obese animals (*F* = 1.305, *P* = 0.265). Lower losses were found in females (slim 6.9 ± 1.6%, obese 7.4 ± 1.6%) compared to males (slim 11.3 ± 2.9%, obese 9.0 ± 1.8%; *F* = 15.377, *P* = 0.001). The regaining of body mass within 24 h differed significantly between the groups (*F* = 5.206, *P* = 0.004), with no difference between genders (*F* = 0.706, *P* = 0.410) and only a trend for a higher mass gain in obese animals (*F* = 3.840, *P* = 0.063), a significant effect of therapy (*F* = 8.275, *P* = 0.009) and a significant gender X therapy interaction (*F* = 6.870, *P* = 0.016), indicating that females gained more mass under therapy than males (Fig. 2). Two and three days after the termination of fasting, there were no significant differences in body mass gains between the groups.

### Beta-hydroxybutyrate in blood

**Fig. 3:**
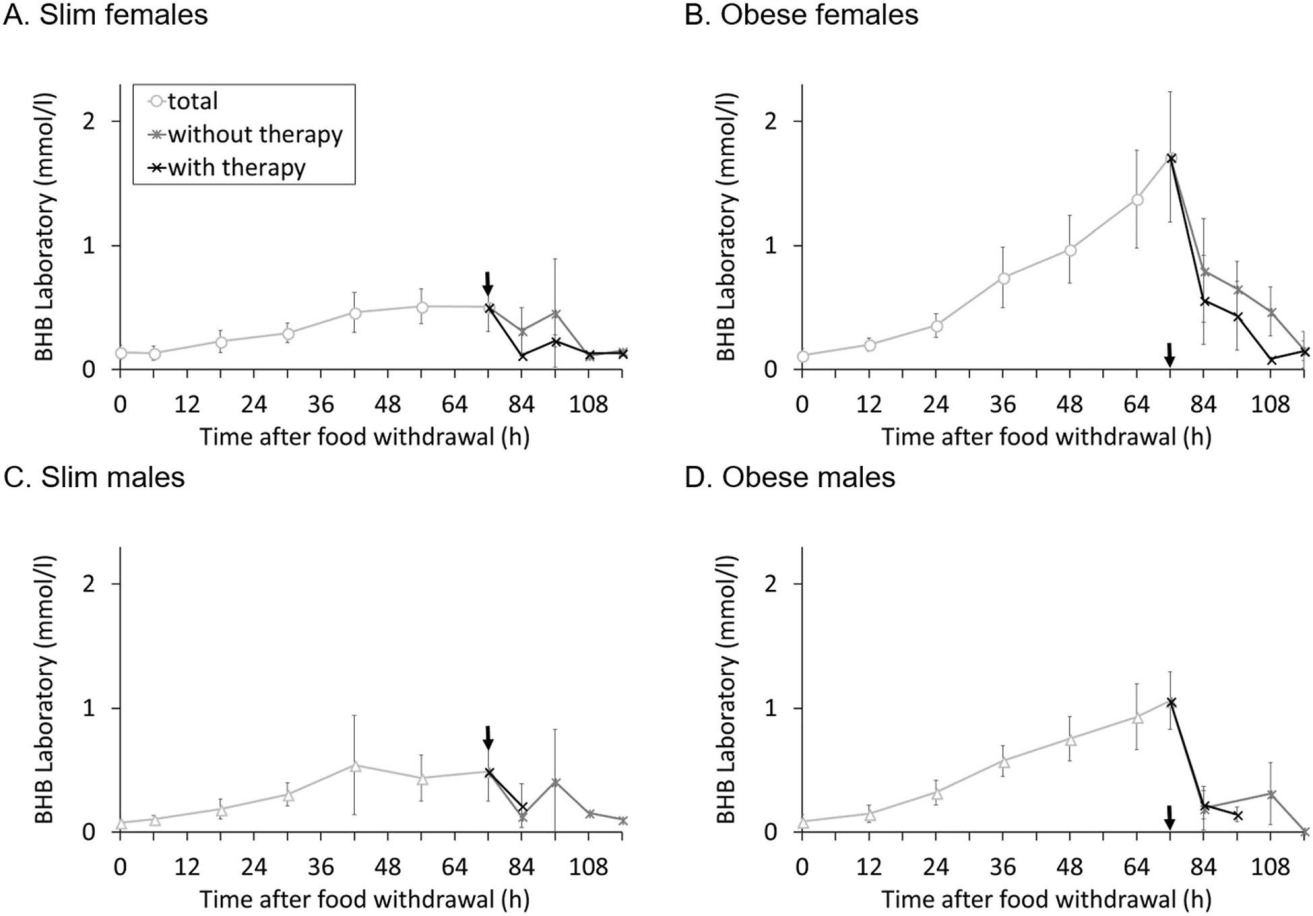
Mean (±SD) β-hydroxybutyrate (mmol/l) in serum (analyzed by the laboratory) of all guinea pigs in one group (*n =* 7, except for slim males *n =* 6); the black arrow indicates the end of the 3 days fasting period. After the end of fasting, animals are divided into treatment groups (*n* = 3/4). **a** Slim females; **b** Obese females; **c** Slim males; **d** Obese males

The elevation of BHB in serum after 3 days of fasting differed significantly between groups (*F* = 21.695, *P* < 0.001) (Fig. 3). Obese guinea pigs had a higher increase than slim ones (*F* = 52.105, *P* < 0.001) and females higher than males (*F* = 5.144, *P* = 0.033). The interaction gender X obesity showed that there was a greater difference in female guinea pigs between the slim and obese ones, compared to the difference between slim males and obese males (*F* = 6.970, *P* = 0.015).

The drop in BHB within the first 12 h of refeeding also differed significantly between the groups (*F* = 8.479, *P* = 0.001), with no effect of gender (*F* = 0.897, *P* = 0.354). However, a clear effect of obesity status (*F* = 21.305, *P* < 0.001) was observed, with obese animals having larger drops, along with a trend for a larger drop in animals receiving therapy (*F* = 3.325, *P* = 0.082). The decline in blood BHB within the first 24 h of refeeding showed a similar pattern, with a significant effect of obesity status (*F* = 24.746, *P* < 0.001) but independent of therapy (*F* = 0.204, *P* = 0.659).

There was a difference between the groups (*F* = 8.308, *P* = 0.001) in the recovery time (defined as the time from the end of fasting until the BHB value decreased to normal levels). Female guinea pigs needed more hours to normalize their ketone levels than males (*F* = 12.021, *P* = 0.002), and so did obese animals compared to slim ones (*F* = 9.213, *P* = 0.006). Animals receiving therapy showed a trend to have a shorter recovery time (*F* = 3.300, *P* = 0.082).

**Fig. 4:**
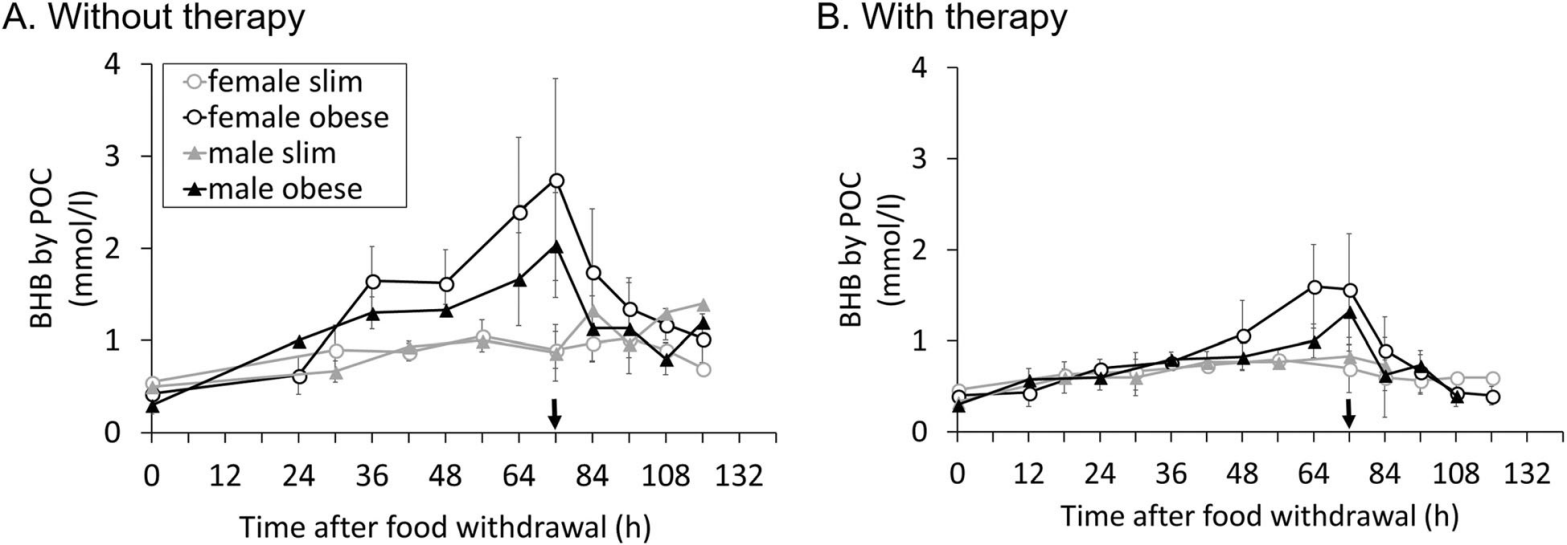
**a** and **b** Mean (±SD) BHB (mmol/l) measured by POC for each group (*n =* 3/4); trends over time during the measurement period, the black arrow indicates the end of the 3 days fasting period. Group II started 9 days after group I

**Fig. 5:**
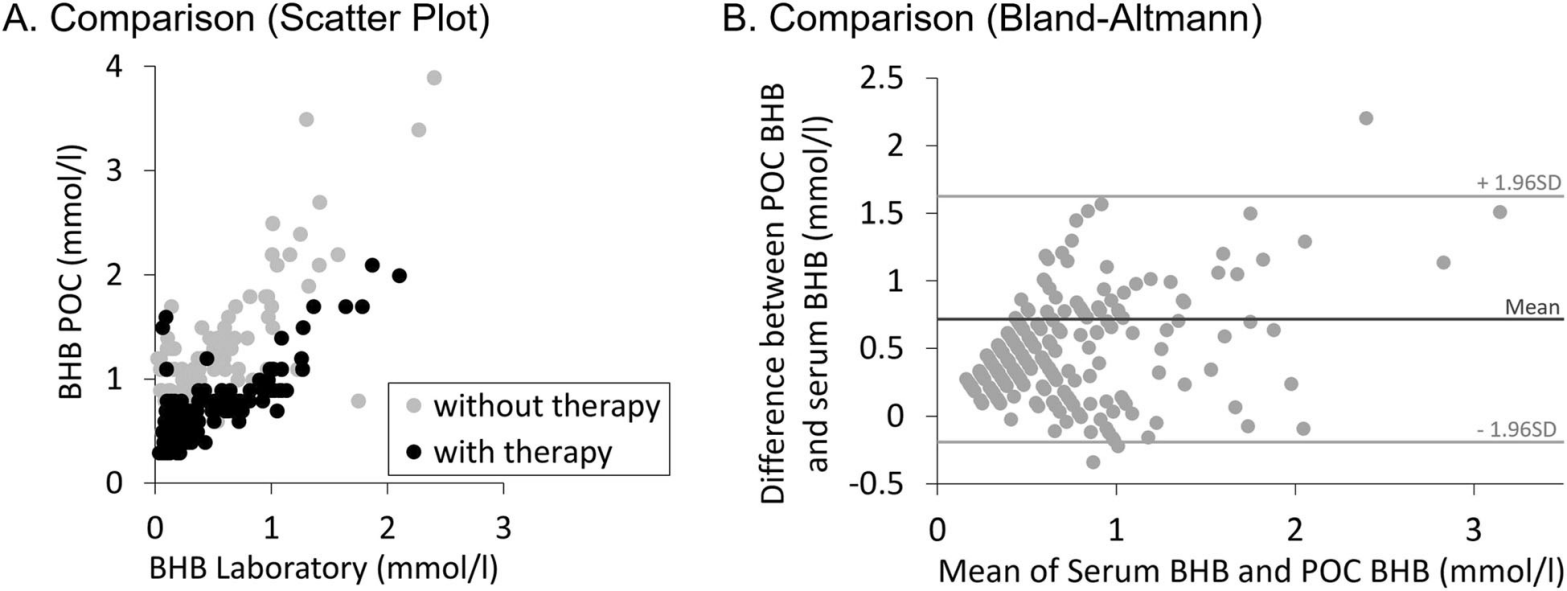
**a** BHB by POC to BHB by Laboratory comparison of Group I (without therapy, *n* = 14) to Group II (with therapy, *n* = 13). **b** Bland-Altmann-Plot to show the relationship between BHB by POC and BHB by Laboratory

While POC BHB data showed similar patterns as BHB measured in the laboratory, there was a systematic offset between the two time periods (Fig. 4). Note that in this case, ‘therapy’ codes for a different time of measurements (9 days difference). The model was significant (*F* = 68.752, *P* < 0.001), with a highly significant correlation between laboratory and POC data (*F* = 153.748, *P* < 0.001). As expected, neither gender (*F* = 0.566, *P* = 0.453) nor obesity status (*F* = 1.899, *P* = 0.170) significantly affected the relationship. However, the time of the experiment, coded by therapy, had highly significant influence (*F* = 92.855, *P* < 0.001) (Fig. 5a).

Additionally, the kappa coefficient was 0 (*n* = 224, *P* < 0.001), indicating no agreement between the two measures. This is also shown in the Bland-Altman analysis (Fig. 5b), which indicates a systematically positive difference, i.e. higher values in serum BHB than in POC BHB. In the corresponding GLM, there was a significant effect of individual (*F* = 4.449, *P* < 0.001), a significant intercept (*F* = 40.192, *P* < 0.001), indicating higher serum BHB than POC BHB values, and a significant slope (*F* = 15.653, *P* < 0.001), indicating that the difference between the two measures increased at higher measurements.

### Ketone bodies in urine

**Fig. 6:**
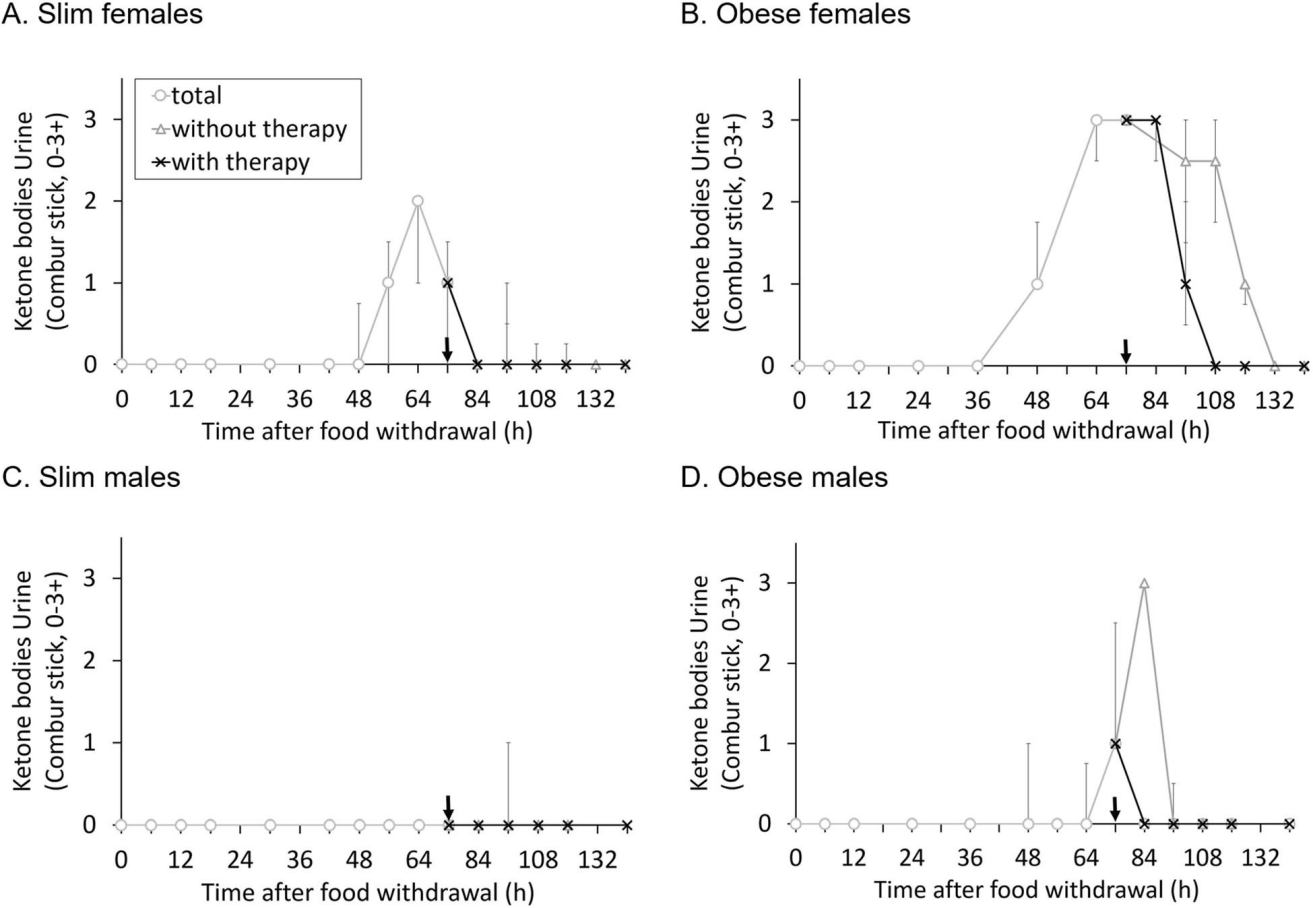
Median (with 1st quartile and 3rd quartile) ketone bodies (semiquantitative, 0–3) detected in urine with Combur stick 9© of all guinea pigs in one group (*n =* 7, except for slim males *n =* 6); the black arrow indicates the end of the 3 days fasting period. After the end of fasting, animals are divided into treatment groups (*n =* 3/4). **a** Slim females; **b** Obese females; **c** Slim males; **d** Obese males

Ketone body levels in urine increased during the fasting period (Fig. 6). Obese guinea pigs showed more intense ketonuria than slim ones (*F* = 19.664, *P* < 0.001) and females more than males (*F* = 5.850, *P* = 0.024). The time from the end of fasting to the normalization of urinary ketone body levels (i.e., levels of 0) also differed significantly between the groups (*F* = 9.874, *P* < 0.001), with obese animals requiring more time to normalize their ketonuria in comparison to the slim ones (*F* = 12.701, *P* = 0.002), females compared to males (*F* = 10.152, *P* = 0.004) or animals not receiving therapy compared to animals receiving therapy (*F* = 6.307, *P* = 0.019).

### *Ketone bodies in urine* versus *Beta***-**hydroxybutyrate in serum

**Fig. 7:**
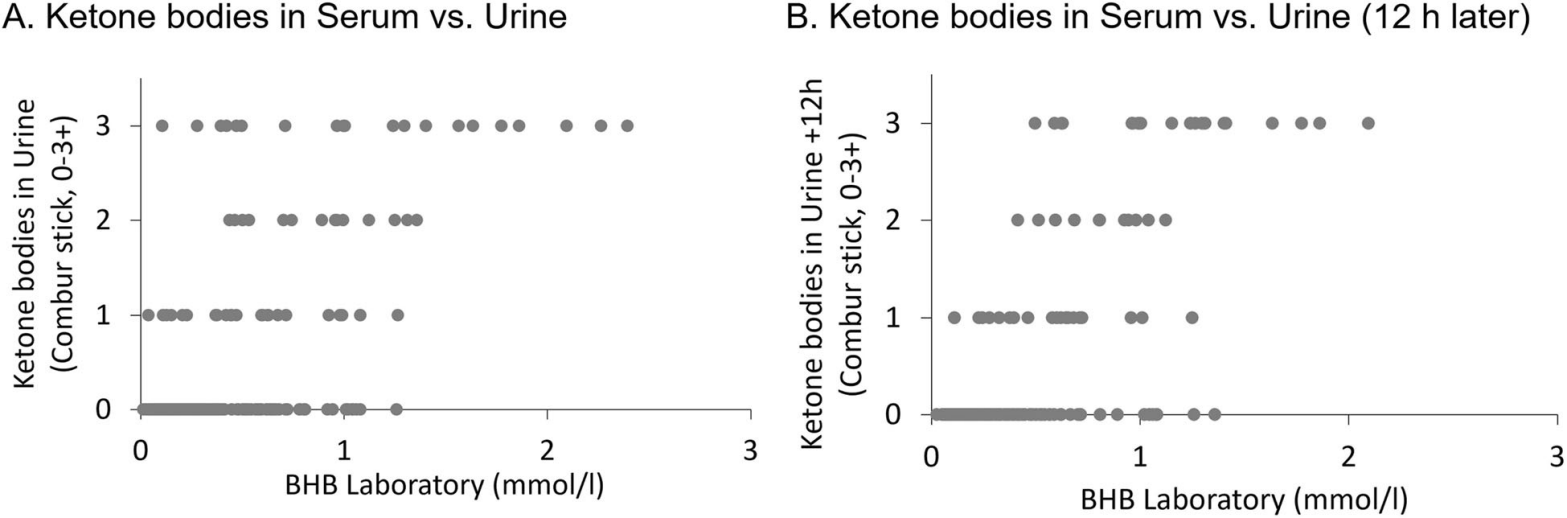
**a** Scatter plot of serum BHB by laboratory compared to ketone bodies in urine by Combur stick. **b** Measurements of BHB in serum compared to measurement of ketone bodies in urine 12 h later

Comparing serum BHB and urine ketone bodies of the same time point and of 12 h later in all cases where all both urine data were available (*n* = 178), there were significant effects of individual (*F* = 1.678, *P* = 0.029 and *F* = 2.136, *P* = 0.002); serum BHB showed a lesser relationship with urine of the same time point (*F* = 43.146, *P* < 0.001) than with urine of 12 h later (*F* = 76.337, *P* < 0.001). (Fig. 7).

### Hematuria

There was significantly more hematuria detected by urinary sticks in samples produced by digital pressure on the bladder than in samples produced spontaneously (chi-square = 6.514, *P* = 0.011).

### Liver to body mass

**Fig. 8:**
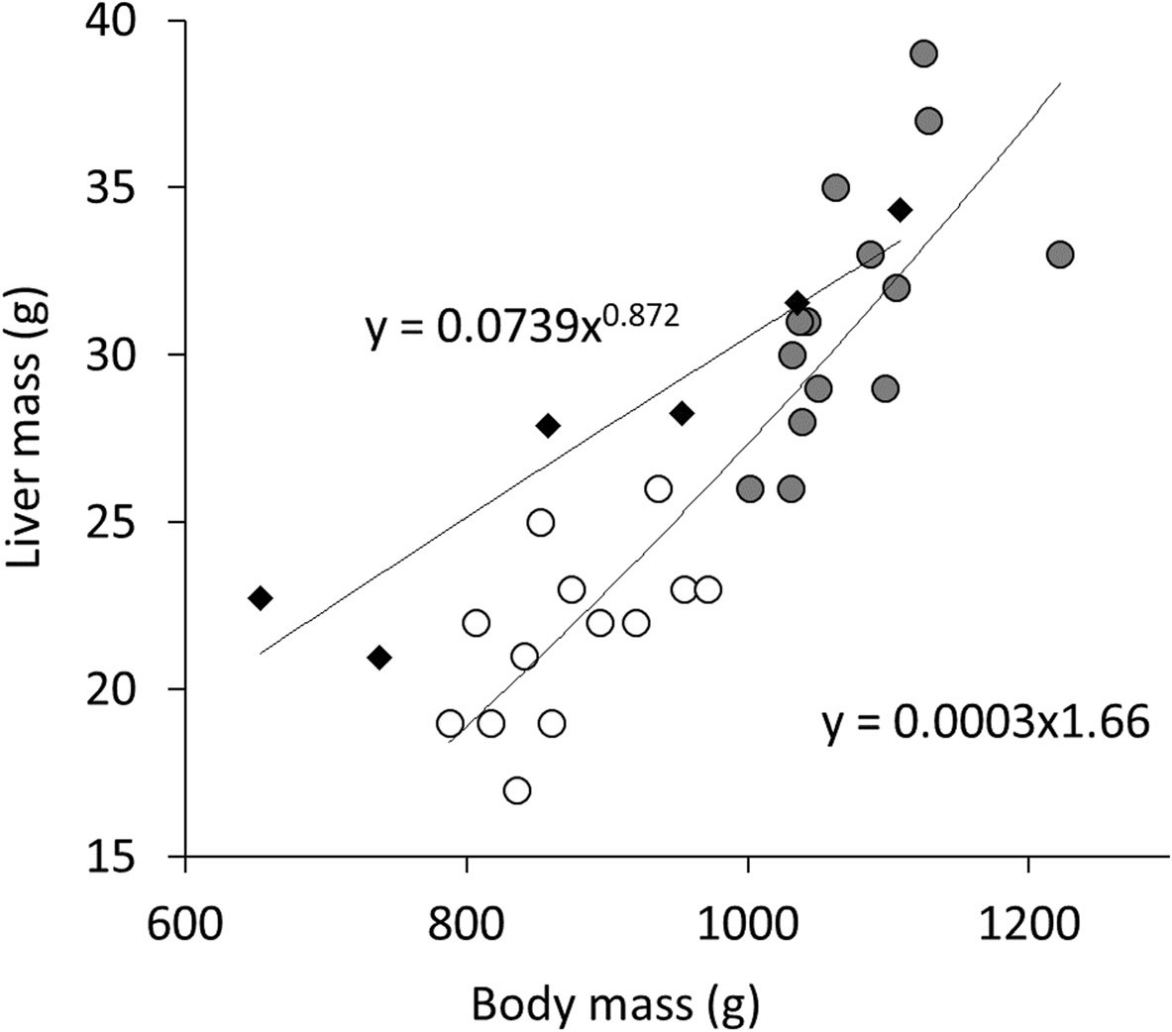
Liver mass (g) in relation to body mass (g) of guinea pigs at time of necropsy after decapitation and bleeding out; our study (grey and white dots, obese and slim animals, respectively) compared to the study by Webster and Liljegren, 1947 [20] (black squares), and the corresponding trendline

Liver mass was significantly related to body mass (*F* = 22.389, *P* < 0.001), with no effect of gender (F = 1.291, *P* = 0.268), obesity status (*F* = 2.056, *P* = 0.166), or therapy (*F* = 0.004, *P* = 0.953). Liver mass scaled to 0.0003 [0;0.0029] BM^1.66[1.32;1.99]^. When assessing slim and obese animals separately, the corresponding equation was 0.0150 [0;11.4025] BM^1.07[0.09;2.05]^ for slim and 0.0009 [0;2.2542] BM^1.50[0.38;2.62]^ for obese animals (Fig. 8).

### Liver histology and laboratory values

The lipidosis score was not affected by gender, obesity status, or therapy. Only the gender X therapy interaction was significant (Tables 1 and 2). The degeneration score was not affected by gender but strongly affected by obesity status and by therapy, with a significant therapy x obesity status interaction (Tables 1 and 2). More degeneration was seen in obese compared to slim guinea pigs as well as in animals with versus those without therapy. The interaction represents an effect of therapy in obese animals, showing more severe degeneration with therapy; this was not seen in slim animals. The same significances were seen for glycogen content score, with an additional effect of gender, indicating a higher glycogen content in males than in females (Tables 1 and 2). The interaction confirmed a higher glycogen content in livers of obese animals with therapy compared to obese animals without therapy. A similar pattern was observed in slim animals, albeit not as distinct. The total liver damage score was only affected by obesity status, indicating increased liver damage in obese animals (Table 1), as shown in the electron microscopy images in the Additional file 1. Affected livers evidence hepatocellular cytoplasmic disintegration, aggregation of electron dense mitochondria with condensed matrices, indistinct cell borders, and condensed nuclear chromatin.

**Table 1:** Statistical data of liver histology scores and laboratory values, comparison between the different groups of guinea pigs (^a^ranked data). See Additional file 1 for descriptive statistics

	Gender	Obesity status (slim/obese)	Therapy	Interaction
	*F* (*P*)			
Lipidosis score^a^	0.203 (0.656)	0.799 (0.381)	0.203 (0.656)	gender X therapy: **4.536 (0.045)**
Degeneration score^a^	2.031 (0.168)	**137.296 (< 0.001)**	**4.619 (0.043)**	therapy X obesity status: **7.427 (0.012)**
Glycogen content score^a^	**4.409 (0.047)**	**98.130 (< 0.001)**	**10.423 (0.004)**	therapy X obesity status: **4.521 (0.045)**
Total Liver damage score^a^	0.826 (0.373)	**34.760 (< 0.001)**	2.053 (0.165)	–
Bile acids	2.981 (0.099)	**5.366 (0.031)**	**10.646 (0.004)**	gender X therapy: **7.885 (0.011)**
ALT^a^	2.222 (0.150)	1.379 (0.252)	**5.186 (0.032)**	–

Significant effects are highlighted in boldface

**Table 2:** Median (with 1st quartile and 3rd quartile) of liver histological scoring (0–9) of Glycogen content, Lipid content and Degeneration score for the different groups of guinea pigs (female/male, slim/obese, with/without therapy)

		Female				Male			
		Without therapy		With therapy		Without therapy		With therapy	
		Slim	Obese	Slim	Obese	Slim	Obese	Slim	Obese
Glycogen content	Median	1.4	4.1	1.5	6.3	1.2	4.5	2.3	7.5
	1st quartile	0.9	3.6	0.9	5.3	1.1	4.4	2	6.9
	3rd quartile	2	4.5	2.1	6.6	1.8	5	2.5	7.8
Lipid content	Median	1.5	1.0	0.8	0.5	0.5	0.8	1.3	0.8
	1st quartile	0.9	0.7	0.6	0.3	0.3	0.6	0.9	0.2
	3rd quartile	2.4	1.5	1.1	0.5	0.8	0.9	1.6	1.8
Degeneration score	Median	0	2.1	0	3.5	0	1.8	0	2.5
	1st quartile	0	1.8	0	3.4	0	1.4	0	2.2
	3rd quartile	0.1	2.3	0.1	3.5	0.1	2	0	3.4

ALT values were higher in treated animals despite that all but two animals were within the reference range (Table 1). Bile acids were highly affected by obesity status and therapy, with a significant interaction of gender X therapy. They showed higher values in obese animals and animals receiving therapy (Table 1).

**Fig. 9:**
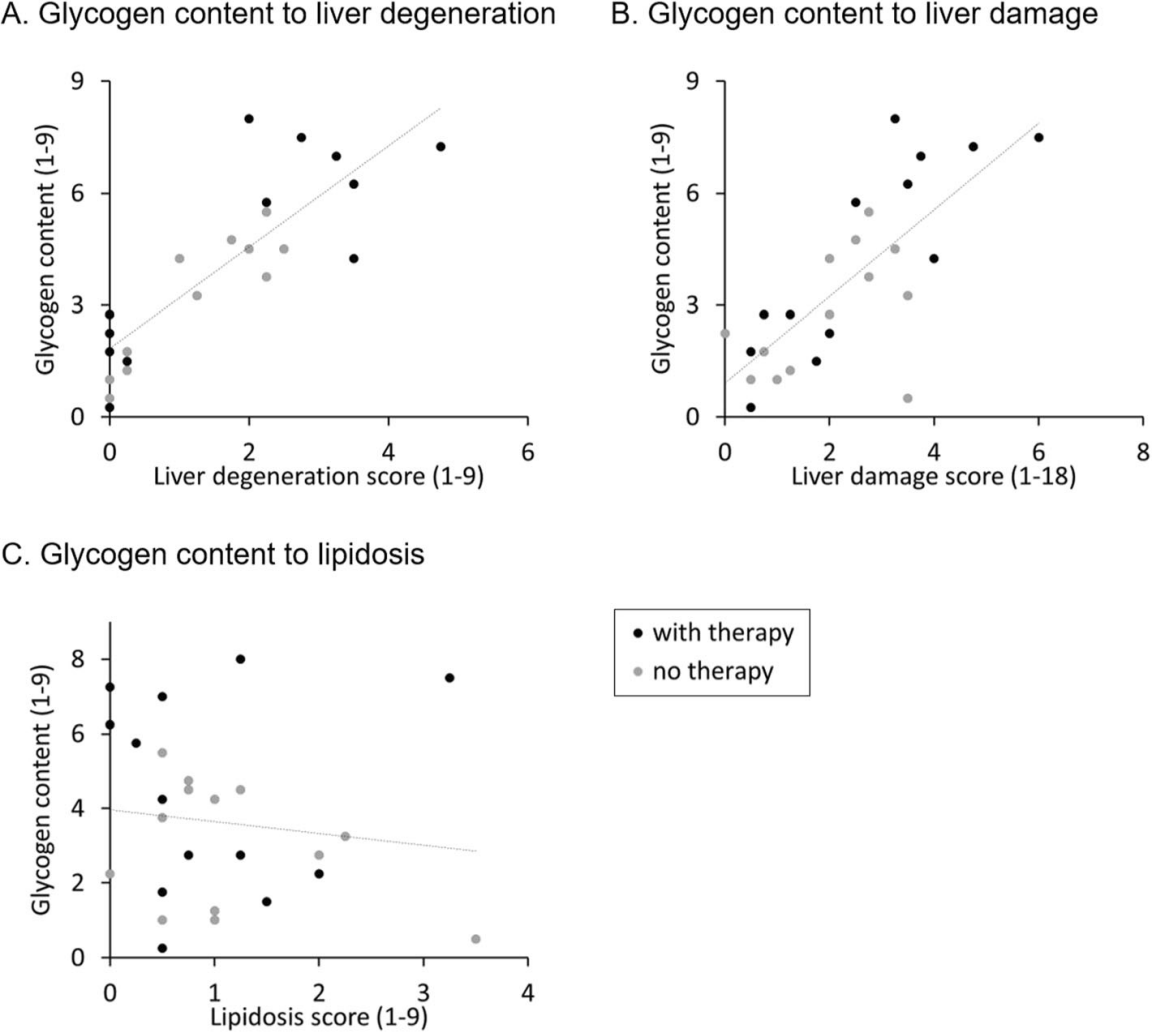
Correlation between glycogen content to other histological changes. Each dot represents the histological score of an individual guinea pig. **a **glycogen content in comparison to liver degeneration; **b** glycogen content in comparison to liver damage;** c** glycogen content in comparison to lipidosis

**Fig. 10:**
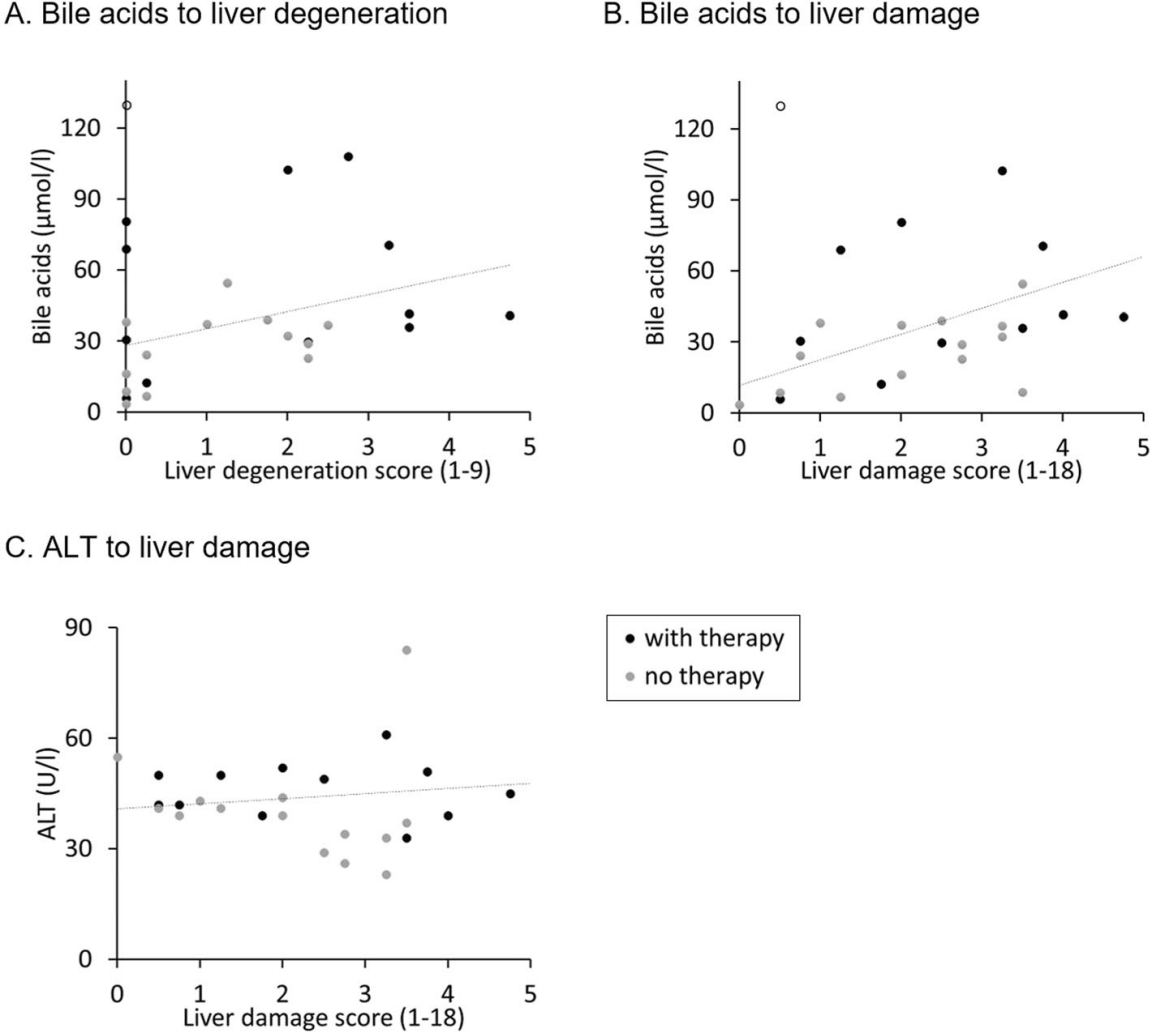
Correlation between different serum parameters to histological changes. Each dot represents one value of an individual guinea pig. The white dot represents an outlier, not included in the statistics due to the reason explained in chapter ‘material and methods’. **a** bile acids in comparison to liver degeneration; **b** bile acids in comparison to liver damage; **c** ALT in comparison to liver damage

Additionally, there was a significant correlation between the glycogen content and the liver degeneration score (*ρ* = 0.83, *P* < 0.001, *n* = 26) and the liver damage score (*ρ* = 0.71, *P* < 0.001, *n* = 26) (Fig. 9a and b), as well as between serum bile acids and the liver degeneration score (*ρ* = 0.44, *P* = 0.026, *n* = 26) and the liver damage score (*ρ* = 0.59, *P* = 0.002, *n* = 26) (Fig. 10a and b). In contrast, the liver enzyme ALT did not correlate with the liver damage score (*ρ* = − 0.06, *P* = 0.790, *n* = 26) (Fig. 10c), and neither did the score of lipidosis to glycogen content (*ρ* = − 0.20, *P* = 0.337, *n* = 26) (Fig. 9c).

## Discussion

The present study focused on the development of acute fasting ketosis in guinea pigs, predisposing factors, diagnostic tools and therapy attempts. We corroborated that intact females seem to be more severely affected by ketosis compared to spayed males, as well as obesity as a predisposing factor for this metabolic disorder, and for longer recovery times thereafter. A positive effect of the implemented therapy protocol with glucose could be demonstrated through improving several clinical indicators of recovery, but it also caused hepatocyte pathology. An additional finding was the evaluation of the POC instrument “Freestyle Precision Neo” for guinea pigs. Although the POC values indicated a similar course of changes as the laboratory data, the correspondence of the two methods was poor. However, an accurate validation that follows the ASCVP-guidelines would need more than just a comparison between two tests.

Generally, the current study only provoked a subclinical ketosis. A stronger reaction, with more distinct differences between groups, would have required a more prolonged fasting period.

### Behavioral changes

Coprophagy, as observed in all animals of the present study, is a normal behavior performed by several small mammals including guinea pigs. The ingested feces had no changes in appearance to normal excrement. Both behaviors, direct and indirect coprophagy, were described previously [21]. Alopecia as a cause of trichophagia is a known issue if nutritional supply is quantitatively or qualitatively unsatisfying [22, 23]. However, trichophagia as a result of environmental stress must also be considered. Only one animal in the present study showed trichophagia, even though all animals were fasted for 3 days and a higher prevalence had been expected.

### Hematuria

Urine collection through digital pressure on the bladder led to more hematuria than collecting spontaneous urinary samples. Nevertheless, 30% of the animals with spontaneous urination showed amounts of blood in urine, and half of the animals where the bladder was emptied through manipulation did not show signs of hematuria. Note that the Combur© stick does not differentiate between Hemoglobin and Myoglobin. The hematuria can be explained by traumatic microlesions in the urinary tract caused by the forced emptying of the bladder. Findings of the present study agree to formerly observed urinalysis, where less blood was seen with spontaneous urination [18].

### BHB by POC

The accuracy of POC measurements in the current study was less precise than reported in other studies for various animal species. In a recent meta-analysis of 18 studies on the diagnostic accuracy of POC instruments for the detection of ketone bodies, an excellent accuracy of Precision Xtra™ for the use in cattle was reported [24]. Additionally, in a fact sheet by Oetzel and McGuirk [25] it was suggested to set the threshold for diagnosis of ketosis with POC values a little lower, because the hand-held ketone meter gave slightly lower test results than the laboratory. Another POC instrument, Precision Xceed® by Abbott® was validated with studies on sheep and cats, showing close correlation with the laboratory reference method [26, 27]. In dogs, an overestimation of BHB concentrations by POC measurement was seen; however, a positive correlation to the laboratory values led to the conclusion that this POC was a useful tool in assessing ketonemia [28].

The present study shows a similar pattern of changes over time in measurements by POC and the laboratory method, but the agreement between the two methods was low, with POC presenting higher values, with the difference increasing at increasing BHB concentrations. Additionally, a generally higher difference between the methods was documented for the group without therapy. A difference of 9 days lay between the two treatment groups, as explained in the material and methods section, and great effort was put into establishing the same environmental conditions: room temperature fluctuated only around ±2 °C with a similar average temperature in both groups. Regrettably, no calibration of the instrument was made ahead of the measurement period, as it was not considered necessary by the manufacturer. This might have contributed to the inaccuracy. Nevertheless, we find that the changes in BHB can be displayed adequately by using the POC, which makes it a suitable tool to interpret a trend. For a single time point value, BHB as determined by laboratory methods appear as the safer option.

### Body mass

Surprisingly, only the females in the obese group gained a substantial amount of body mass (86.3 g ±61.5; 8.2 ± 5.9%) during the feeding period, whereas the females in the slim group just barely kept theirs. In contrast, both male groups gained a similar amount (45.4 g ±52.6 vs. 51.5 g ±35.8; 4.4 ± 5.2% vs. 5.9 ± 4.1% for the obese and slim groups respectively), which was roughly half of the obese females’ body mass gain. Considering the impact of castration and the stressful condition preceding it, one could argue that the males regained only the body mass already lost due to stress. Nonetheless, we expected a greater increase in body mass of high energy-fed animals, as seen in Lachmann et al. [4]. Pitts [29] found that female guinea pigs have a greater capacity to store fat in comparison to males, which could explain the difference seen between the obese females and obese males in the current study.

The body mass loss of 6.9 ± 1.6% to 11.3 ± 2.9% after 3 days of fasting reported in the present study is lower than the documented in former studies, where losses of 12 to 25.5 ± 1.8% within 3 to 4 days were described [4, 6, 9, 30]. A great proportion of the body mass loss during fasting is presumably the loss of ingesta from the digestive tract as discussed in Bergmann and Sellers [6]. In the present study, guinea pigs were observed to perform coprophagy on a regular basis, which could explain the less severe body mass loss. On the other hand, most of the former studies used young animals, still in growth, and therefore of lesser body mass to start with, and higher energy requirements, which could have led to a greater impact of starvation.

Within the first 24 h after refeeding, a trend in greater mass gain of obese animals was seen. As the guinea pigs were initially separated into groups according to their arrival body mass it is thereby possible they were indirectly also selected for their tendency to gain mass, determined by genetic or epigenetic factors. Additionally, female guinea pigs under therapy gained significantly more body mass on day 1 compared to the male group receiving therapy. However, after day 2 no difference was seen anymore. To what degree the results of the castrated males of the present study can be transferred to intact males remains to be investigated.

### Development and regression of ketosis

According to Kraft et al. [15], healthy animals do not excrete any ketone bodies in urine and their blood level of BHB is less than 0.6 mmol/l. In cows, the threshold for subclinical ketosis is set at 0.9–1.7 mmol/l BHB in serum; for a value above 1.7 mmol/l BHB in serum, clinical manifestation is to be expected. Looking at values obtained herein, the threshold might be similar. Yet, the study did not allow to define a threshold for subclinical or clinical ketosis, since no clinical signs were observed. Additionally, pregnant guinea pigs might be more susceptible and have a lower threshold, because in Lachmann, et al. [4] obese pregnant females had lower average BHB values and became severely ill, likewise in Ganaway and Allen [9] where non-pregnant obese female guinea pigs showed signs of ketosis but not as severely as pregnant ones. Probably, more time than in our experiment is needed until animals become clinically ill: In Lachmann, et al. [4] nonpregnant females and males started to show signs of illness after 4 days of fasting. Further, the different onset of a clinical disease might be explained by the fact that pregnant animals have higher energetic requirements, animals developing ketosis as a secondary problem may be weak already due to the primary disease, and might also be unable to perform coprophagy, which could delay the process. However, these hypotheses requires further investigation.

As suspected, obese guinea pigs showed a higher susceptibility to develop a metabolic imbalance while fasting compared to slim ones. After refeeding, the obese animals needed more time until BHB values decreased to normal levels and ketone bodies were eliminated from urine. A study by Ganaway and Allen [9] also induced higher serum BHB in adipose animals and reported an outcome in non-pregnant guinea pigs after fasting similar to the one observed in the current study. This is explained by the greater fat storage of high energy fed animals prior to fasting. Therefore, more fat is mobilized in an anorexic stage and transported to the hepatocytes, where an excessive supply leads to ketone body production and hepatic lipidosis [4, 6, 8, 19]. Additionally, the impact of insulin should be discussed, as it has an antilipolytic effect. Obese animals can develop insulin resistance, leading to higher lipolysis and consequently more ketogenesis and lipidosis than leaner individuals [31, 32].

Fasting seems to have a greater impact on female animals than males. In the current study, a greater difference in females between the obese and the slim group compared to the equivalent male groups was seen. The study findings agree with Butts and Deuel Jr. [33], who found that female guinea pigs excreted twice the amount of acetone bodies than their male counterpart after administration of acetoacetic acid. The authors relate this disparity to gender differences in the ability to oxidize acetoacetic acid, and claim a higher susceptibility to ketosis for female individuals. No blood parameters were measured in that study. In contrast, the study by Lachmann et al. [4] found male guinea pigs to excrete more ketone bodies in urine and form higher BHB peak values in blood than females after 4 days of fasting (BHB values of males: 1.40 ± 0.39 mmol/l; females: 0.83 ± 0.69 mmol/l [4]). However, only acetone in urine was measured in their study, whereas in the current experiment both acetone and acetoacetate were measured. Besides, the male guinea pigs in the study by Lachmann, et al. [4] were heavier in the beginning of the fasting period and perhaps more obese than the females (body mass of males: 1030 ± 175 g; females: 855 ± 131 g [4]). Obesity may be a decisive factor, and differences between studies could then be explained through the difference in body mass of the male and female individuals.

Additionally, the stage of estrous cycle was undetermined in the female used in the current study, which did not allow to determine the existence of a stage effect on the development of ketosis, as has been discussed in dairy cows [34].

After the end of the fasting period, females required more time to normalize their BHB levels in serum and to cease their ketone body excretion in urine. Bacchus et al. [13] injected BHB intraperitoneally into young female guinea pigs, determined the total ketone body concentration in blood through measuring acetone, and reported a half-life time of 68 (±2.1) min. Contrastingly, in the current study, considering the final BHB values at the end of the fasting period and the next subsequent BHB measurement after 12 h, the theoretical half-life time of values would be about 6 to 12 h. The present study did not allow to define an accurate half-life time, because the measurement intervals were not sufficiently frequent, and we have to assume that BHB production did not cease immediately with refeeding. Therefore, the difference between studies seems reasonable, as the animals in Bacchus’ study [13] were healthy and only had to eliminate the injected BHB, whereas our animals produced BHB by themselves, and had to down-regulate its production in parallel to eliminating the product.

### Effects of therapy on the regression of ketosis

Positive effects of therapy versus no therapy, i.e. additional glucose injection in contrast to merely refeeding, were observed. First, a trend for larger drops in BHB levels within the first 12 h after fasting was recorded. Secondly, those animals receiving therapy tended to have a shorter recovery time considering BHB level decrease, as well as urine ketone body elimination. Studies in rats on fasting ketosis by Foster [35] described an abrupt cessation of ketone body production by the liver after intravenous administration of 0.3 ml glucose 50%, a decline of acetoacetate beginning within 5 min. This immediate decrease in serum acetoacetate concentration was too large to be accounted for by a simple dilution effect. Moreover, tube feeding of 5 ml high glucose diet led to a reversal of ketosis within 15 min, inducing the same metabolic effect. This agrees with findings from the current study, but occurring much faster and explained by the more intense intervention in those experiments.

### Comparison of urine to serum ketone body remission

The initial hypothesis in the current study was that ketone bodies in blood would disappear earlier than in urine, which we could not confirm. Ketone bodies detected by urinary dip stick are only acetoacetate and acetone, but the greatest fraction of ketone bodies in fasting ketosis is usually BHB at 80%, and a change in color of the urine test stick is only detectable when ketone bodies exceed a certain concentration in urine [15]. However, in the ketone body cascade in direction of ketone body reduction, BHB is metabolized to acetoacetate and further to acetyl-CoA, which is being integrated into the citric acid circle if enough oxaloacetate is available, or alternatively reversed to the fat storage as triglycerides. This would mean that urinary dip sticks do not represent the full extent of the disease in the fasting stage. Considering the ketone body cascade, the assumption can be made that acetoacetate degrades as the latest of all ketone bodies and is a good indicator of ketosis remission. Comparing urine ketone bodies and BHB in serum, there was a better correlation with measurements of serum and urine 12 h later; therefore, the current trend of ketone bodies is more accurately shown in blood and represented in urine with some time difference.

### Liver mass to body mass

The liver mass in comparison to body mass has an unusual scaling of y = 0.0003 × ^1.66^. Normal liver mass to body mass was described by Webster and Liljegren [20], where they measured different organs of guinea pigs. The trendline of their values shows a gradient of y = 0.0739 × ^0.87^ (Fig. 8). This matches the statement by Rocha et al. [36] that liver mass is aligned with the overall organism’s metabolism. Results gathered herein clearly deviate from these findings. The exponent found by Webster and Liljegren [20] was included in the 95% confidence interval of the slim animals; even though the scaling exponent for liver mass did not differ significantly between slim and obese animals in the present study (due to overlapping 95% confidence intervals), the scaling was steeper in the obese specimens, suggesting that the overall extreme scaling in animals derived from a pathological condition of the liver due to fasting that was particularly pronounced in this group.

### Histological findings of the liver

Guinea pigs suffering from ketosis showed very fatty livers at necropsy, and the livers were 10% heavier than the ones of control animals [6]. In the current study, no significant difference of lipidosis between the obese and the slim guinea pigs was documented. Females receiving therapy showed less hepatic lipidosis than the ones not treated, and the opposite outcome was seen in males, where the treated animals showed more signs of lipidosis. Several other studies found severe fatty livers in obese guinea pigs following an anorexic period, reporting higher lipid content in the liver of obese animals versus those of a normal body condition and more in livers of ketonic guinea pigs than healthy ones [3, 4, 8, 19, 37–39]. These findings were all documented directly after the fasting period, when highest fat mobilization was in progress. Evaluation of the liver in the present study was performed 1 week after refeeding, probably explaining the difference in outcome. Nevertheless, obese animals probably mobilized more fat to the liver while fasting, and therefore more hepatic degeneration was seen in obese animals compared to slim ones at the end of the study.

Higher glycogen content was seen in the male liver in comparison to females, confirming previous findings [40], although no higher lipid content was seen in female livers herein. Foster [35] described a negative correlation between the lipid and glycogen content of the liver, with glycogen declining while fat content increased during fasting. Similar findings are shown in Bergman and Sellers [6]. No correlation was seen at the timepoint of measurements in the current study, and no trend over time was recorded. Nevertheless, we saw obese guinea pigs to have significantly higher glycogen contents in the liver in comparison to slim animals, probably due to differences in the diet. The obese group on an energy-dense diet was supplied with hay and a mixed grain feed ad libitum whereas the slim groups only had hay and grass at their disposal. Fréminet [30] described hepatic glycogen content in rats and guinea pigs to be almost exhausted within 24 h of food deprivation and remaining low until 96 h of fasting; after refeeding, the hepatic glycogen content exceeded the one of the control animals.

Another predictable difference was between the guinea pigs with and those without therapy. Those having had fluid and glucose injection showed more glycogen in their liver. The injected glucose is primarily oxidized directly for energy, and the remaining glucose in depleted animals transforms predominantly toward hepatic glycogen [41]. Additionally, we revealed a correlation between glycogen content and damage of the liver. As described in Fréminet [30] depleted animals are more likely to store additional glucose as hepatic glycogen. Excessive glucose substitution could have led to a greater impact on the liver through a glucose overload, with the following storage as hepatic glycogen and consequently a delay of regeneration from hepatic lipidosis. This might be an explanation for the greater hepatocyte degeneration seen in guinea pigs receiving therapy. Probably, an initial dose of glucose to stop ketogenesis is beneficial. Afterwards, the animal should be observed carefully, and glucose therapy only continued if the animal does not start eating on its own.

### Laboratory parameters

In vivo testing of serum parameters to evaluate liver alterations is a less invasive method than taking biopsies for histology. Therefore, ALT and bile acids were measured in the present study to evaluate their usefulness in guinea pigs. ALT is relatively specific to liver in rats and an accepted biomarker for the detection of liver injury in preclinical models [42–44]. However, this liver enzyme is not convincingly associated with histopathological findings [45, 46]. This stands in agreement with findings of the present study: no values outside the reference range were found and no correlation to the liver damage score documented, which makes ALT a non-reliable parameter for liver injury in guinea pigs. Contrastingly, bile acids showed a significant correlation to the liver damage score. Higher bile acid levels were seen in obese animals, reflecting overall finding of obese guinea pigs being more severely affected by the impact of fasting. Bile acids have been described in various species as associated with liver diseases, hepatic damage or fatty liver [46–49]. A drawback of the total bile acids is that they are only a sensitive indicator for an overall assessment of hepatic damage but give little insight in the specific damage or pathogenesis. The changes in bile acids suggest a decrease in liver function; therefore, it would be interesting to see whether other liver function parameters such as total protein, fibrinogen, urea and clotting factors change during ketosis, too. Additionally, it might be worthwhile testing the use of urine for bile acid screening [50] in guinea pigs as they are an easily stressed species if handled too intensively.

## Conclusions

An anorexic state in guinea pigs should be considered as a serious condition, likely leading to death if initiation of treatment fails. It is therefore essential that those cases are treated immediately to reverse the katabolic state they are usually in at the time of presentation. We tested an initial supportive fluid therapy with glucose supplementation to terminate the production of ketone bodies and reduce the metabolic imbalance. As an additional benefit, we saw that resolution of the metabolic disorder seems faster when supportive therapy is provided. However, the recommended subsequent fluid therapy should be without additional glucose as it seems to alter the recovery of the liver. To follow the trend of ketosis and the change in metabolic condition of the animal the urinary dip stick presents itself as a valuable tool to detect trends. Measuring the bile acids seems a helpful value to detect an impact on the liver and to estimate histological damage. Further studies which create a more intense metabolic imbalance through fasting for longer time periods are suggested to investigate clinical manifestations of ketosis. As an addition, liver values in blood (bile acids and ALT) should also be measured during the fasting and refeeding period instead of at the end of the experiment; this would ensure a more accurate evaluation of liver parameters.

## Methods

### Animals and housing

Fourteen clinically healthy male and female adult guinea pigs each (strain Dunkin Hartley HsdDhl:DH) were used in this study. All the animals were retired breeders from Envigo RMS (B.V., The Netherlands), and were aged between one and 2 years old. In the female guinea pigs the stage of estrous cycle was unknown. Upon arrival, the animals were divided according to their body mass into four groups with seven animals each (slim and obese females, and slim and obese males). The initial body mass of slim females was 963 ± 45 g, of obese females 1058 ± 29 g, of slim males 867 ± 35 g, and obese males 1071 ± 71 g. All animals were submitted to a general health check with special focus on their teeth to ensure a clinically healthy dentition. The female guinea pigs stayed intact, whereas the male guinea pigs were castrated to facilitate group husbandry [12]. Castration was performed by surgical orchiectomy, 6.5 weeks before to the beginning of the experimental period. The 2 weeks after surgery were excluded from the observational period of the study. The study consisted of a feeding and observation period of 59 to 68 days spent in an outside group enclosure, an experimental period of 9 days in individual indoor cages (3 days adaptation to the indoor cages, 3 days fasting, 3 days re-feeding, with or without replacement fluid therapy) and again a final observation period in the outside group enclosures of 6 days.

The outside enclosures for each of the two slim groups was 4.45 m in length and 1.12 m in width (approximately 5 m^2^). About two thirds of this area was covered by grass, and one third with a substrate of cleaned sand of 1–4 mm in grain size. The enclosure for each of the two obese groups was 2 m in length and 1.12 m in width (2.2 m^2^), and the whole area was covered with sand with no access to grass. Outside enclosures were protected against rain and direct sun. All groups had a variety of shelters at their disposal, whose floors were filled with wood shavings.

The individual indoor cages had a ground area of 0.74 m^2^ per animal. Opportunity for contact with other individuals was provided by holes in the side walls of the enclosures. The males were kept in one room and the females in another. Every cage had an elevated platform and a shelter. Apple tree branches were offered as gnawing material to all animals in the outside and inside enclosures. Wood shavings were used as litter during the adaptation and treatment period. For the 3 days of fasting, the litter was changed to sand, to avoid pica behavior.

### Feeding

The slim group was fed with grass hay ad libitum and the fresh grass that grew in the enclosure. To ensure a steady regrowth of the fresh grass, a certain portion of the grassy area was always fenced off on a rotating basis. The obese group was fed with grass hay (50 g/animal and day) and a mixed grain feed^3^ for guinea pigs (40 g/animal and day). The mixed grain feed had the following ingredients: wheat, oats, barley, corn, peanuts, sunflower seeds, pellets with herbs, vitamins and minerals. Vitamin C supplementation was administered to all groups by 200 mg ascorbic acid per 1 L fresh water [51, 52]. Each group had both nipple drinkers and water bowls in the outside enclosure. During single housing, every animal had two nipple drinkers. Water was provided for ad libitum intake at all times.

### Animal experiment

This experiment was approved by the Animal Care and Use Committee of the Veterinary Office of Zurich (Nr. 27,368, ZH003/16). The animals were fed as described above during the feeding period to either keep their slim body condition or to become obese. Additionally, to evaluate the effect of therapy on the course of ketone body excretion and liver histology, the animals were ascribed randomly a priori to a therapy or a non-therapy group. The animal experiment license contained the legal obligation to treat any animal that would show clinical signs of ketosis (defined as anorexia after refeeding, ruffled hair, respiratory distress, depression, lethargy, apathy, somnolence, prostration, convulsions, muscle spasms, paralysis, coma or death), with the same therapy as intended for the ‘therapy group’.

During the feeding period animals were weighed once a week and daily health checks were made, consisting of observing changes in posture, fur quality, mobility, breathing, group interaction (isolation of group members), external injuries, ocular or nasal discharge, and cleanness of the anal region. Palpation of the abdomen and evaluation of oral and ocular mucosal membrane was performed during weekly weighing. In this period, one slim male animal had an ocular injury (perforated infected corneal ulcer) and had to be treated according to the ophthalmologists’ instructions for 14 days. This animal later occurred as an outlier in the bile acid measurements and was excluded from statistical evaluation.

Another slim male showed a chronic mass loss and did not improve his condition despite additional force feeding with Oxbow’s critical care™,^4^ and had to be euthanized following the ethical criteria of the study. The animal was anesthetized with isoflurane administered by face mask and subsequently injected intracardially with 200 mg/kg pentobarbital (Esconarkon^5^). The necropsy findings were a reduced body mass, diffuse hepatic lipidosis, mild interstitial calcification of the kidney and an alveolar lung edema. No signs of infectious diseases were reported.

During the 9 days in individual cages, the animals were weighed and submitted to a health check (performed as explained above) in the morning of each day. The start of the 72 h fasting period was set as time point 0, which is also the beginning of the measurement period. All the substrate, food and chewing material was removed from the cages and a sandy substrate was added instead. Fasting started at 8 am. For the refeeding/therapy period, the substrate was changed back to wood shavings and the animals received the normal daily ration of food according to their group (slim/obese). According to their ascribed group, animals were either only fed (no therapy), or additionally treated with two 20 ml subcutaneous injections per day of Ringer Acetate and Glucose 5% (in a ratio of 50:50) for as many days as it took to reach baseline ketone body levels (two to three days).

At time point 0, samples were collected to determine the basal value for ketone bodies in urine as well as BHB in blood for POC and in serum for laboratory analysis. Blood sampling was scheduled subsequently at 72, 84, 96, 108, 120, 132 and 144 h in all animals, and additionally at 6, 18, 30, 42, and 56 h in slim and at 12, 24, 36, 48 and 64 h in obese animals; urine sampling was scheduled for all animals at each of these time points. When urine samples indicated no more ketone bodies an individual animal, only two more subsequent blood samples were taken. The difference in sampling time was due to logistic reasons, as it was always the same observer taking the samples and notes for all the animals. The sampling time for slim and obese animals during fasting were different; this difference ensured that for every timepoint, there was a measurement of at least 3 animals of a group to establish a mean/median. We decided on this layout as we did not want to discuss the different timepoints themselves, but the overall trend of the measurements over time.

Urine samples were preferably taken from spontaneous urination into transport or anesthesia induction boxes, or otherwise by gentle digital compression on the bladder. Blood samples were either taken by venipuncture of alternating sides of the *Vena saphena lateralis* under manual restraint, or from either the right or left *V. cava cranialis* under general isoflurane anesthesia of 2–3 min, induced at 5% isoflurane in an induction box and maintained at 1.5–2.5% isoflurane (at a mixed air and O_2_ flow of 1 L/min) by a face mask. The volume was always 0.3 ml per sample. This resulted in a total removal of nearly 4 ml of blood per animal within 144 h (0.35 to 0.45% of the body mass). Blood samples were centrifuged at 4′000 g for 10 min and serum was pipetted into tubes for laboratory analysis.

The period of individual husbandry including fasting and treatment was done in two batches so that all animals could be evaluated by the same investigator. Due to this sequence, it was decided to first evaluate non-therapy animals in case one of them would develop clinical signs to an extent that required therapy, as requested by the ethical criteria of the study. Because no animal developed clinical illness (see results), this resulted in all animals from the second batch receiving therapy.

For the subsequent observation period, all the animals were returned to their former outside enclosures, in the same groups as before. Their general condition was checked daily for 1 week and body mass assessed twice a week.

### Termination of the study

The animals were euthanized by bolt stunning (Dick KTBG spring-powered, captive-bolt gun^6^) using the method described in Limon et al. [53], without a specific fasting period. The gun was placed at the crossing point of the line between the eyes and ears. Followingly the animals were bled out by cutting the carotid arteries and jugular veins bilaterally. Post mortem blood (mixed venous and arterial blood) was collected immediately for serum bile acid and ALT analysis and the animals weighed subsequently. A necropsy was performed, the liver was removed completely and weighed. Samples of the left lateral, right lateral, and caudal lobes of the liver were collected and fixated into 10% neutral-buffered formalin for histological analyses. The paraffin-embedded tissues were sectioned at 5 μm and stained with hematoxylin-eosin (H&E), Periodic acid-Schiff reaction (PAS) and oil-red staining. A score for liver damage was established (see Additional file 1 for score definition) and ascribed to each of the slides. Three randomly picked representative fields were evaluated in each slide at a magnification × 200. Each of the histological liver lobe parts (periportal (1), intermediate (2) and centrilobular (3)) was scored according to a 3-point scale of lesion severity for degeneration, lipid content, and glycogen content. This led to a maximum total damage score of 27 per animal (3 locations, 3 scores each with a maximum of 3 for each individual score). Transmission electron microscopical tissue samples were fixed in 2.5% glutaraldehyde (EMS) buffered in 0.1 M Na-P-buffer overnight, washed × 3 in 0.1 M buffer, post fixed in 1% osmium tetroxide (Sigma-Aldrich) and dehydrated in ascending concentrations of ethanol, followed by propylene oxide and included in 30 and 50% Epon resin (Sigma-Aldrich).

At least three 0.9 μm thick toluidine blue stained semithin sections per localisation were produced. Representative areas were trimmed and 90 nm, lead citrate (Merck) and uranyl acetate (Merck) contrasted ultrathin sections were produced and viewed under Phillips CM10, operating with Gatan Orius Sc1000 (832) digital camera, Gatan Microscopical Suite, Digital Micrograph, Version 230.540.

### Sample analysis

The point of care instrument (Freestyle Precision Neo)^2^ was used to measure BHB in 1.5 μl of full blood. The POC can read values from 0.0 to 8.0 mmol/L. The laboratory used the BHB LiquiColor® Test^7^ to quantify the amount of BHB in serum with an enzymatic approach.

To analyze urine directly, a urinary dip stick (Combur 9©)^1^ was used.

Alanine Aminotransferase (ALT) activity and total bile acids concentration were measured on an automated chemistry analyzer^8^ using the IFCC method for ALT and an enzymatic method for total bile acids. Two levels of internal quality control samples were measured on a daily basis prior to the patient samples. Furthermore, proficiency testing was performed four times per year.

### Statistical analysis

Data are displayed as means ± standard deviation. Data were analyzed by General Linear Models (GLM; confirming normal distribution of residuals by Kolmogorov-Smirnov-test), with gender, obesity status (slim/obese) and, when appropriate, therapy (without/with) as cofactors; if two-way interactions were not significant, the GLM was repeated without the interactions. For liver mass, body mass was added as a covariable in the GLM. If residuals of a GLM were not normally distributed, or if the nature of the data a priori excluded a parametric test (as in the case of dip stick readings or liver scores), the respective GLMs were performed using ranked data. Because the BHB in POC data were not normally distributed, not even after log-transformation, a General Linear Model with ranked data was performed, comparing POC data (dependent variable) with laboratory data (independent variable), using gender, slim/obese and therapy as co-factors.

In order to assess how serum BHB and POC BHB measurements correspond to each other, we calculated the kappa statistic, and made a Bland-Altman plot, testing the relationship between the mean of the two measures and their difference with a General Linear Model (GLM, confirming normal distribution of residuals), with the difference as the dependent and the mean as the independent variable and individual as random factor (to account for repeated measures).

Moreover to assess whether there was a correlation between serum BHB and urine ketone bodies, we performed two General Linear Models, using ranked data for serum BHB (making the GLM a nonparametric test), with urine ketone bodies as the dependent and serum BHB as the independent variable, and individual as a random factor (again, to account for repeated measures). The GLM was run for measurements made at the same time points, and repeated with measurements of urine 12 h after the serum measurements.

The scaling of liver mass with body mass was assessed by linear regression of log-transformed values, reporting parameter estimates [and their 95% confidence intervals]. The risk of hematuria depending on the method of urine sampling was assessed by chi-square test. Correlations involving non-parametric data were assessed by Spearman’s *ρ*. All analyses were performed in SPSS 23.0 (Statistical Package for the Social Sciences) [54]^9^ with the significance level set to 0.05.

## Supplementary information

**Additional file 1.** Information about the detailed treatment plan of one animal during the feeding period; supplement to table 1 (basic data used for statistics); standard operating procedure (SOP) for the scoring system of the liver histology; electron microscopy images of the liver.

## Data Availability

The datasets supporting the conclusions of this article are available from the corresponding author on reasonable request.

## Abbreviations

ALT: Alanine Aminotransferase
BHB: β-Hydroxybutyrate
GLM: General linear model
IFCC: The International Federation of Clinical Chemistry and Laboratory Medicine
POC: Point of care

## Footnotes

1 Roche Diagnostics GmbH, D-68305 Mannheim, Germany

2 Abbott© Diabetes Care Ltd., Oxon OX29 OYL, UK

3 vita-balance, LANDI Art. 26,267, Landi, CITY, Switzerland

4 Oxbow Animal Health, Omaha, NE 68138 USA

5 Streuli Pharma AG, 8730 Uznach, Switzerland

6 Friedr. Dick GmbH & Co.©, D-73779 Deizisau, Germany

7 interchim®, 03103 Montlaçon Cedex, France

8 Cobas 6000 501, Roche Diagnostics, Rotkreuz, Switzerland

9 IBM, Armonk, New York, USA
