# Supplementary material for: Development, diagnosis and therapy of ketosis in non-gravid and non-lactating Guinea pigs
Source: BMC Vet Res. 2020 Feb 3;16:41. doi: 10.1186/s12917-020-2257-2 (PMC6998326; doi:10.1186/s12917-020-2257-2)
Supplement: Supplementary file 1 — Additional file 1. Information about the detailed treatment plan of one animal during the feeding period; supplement to table 1 (basic data used for statistics); standard operating procedure (SOP) for the scoring system of the liver histology; electron microscopy images of the liver. [file 12917_2020_2257_MOESM1_ESM.pdf]

Supplemental material

Development, diagnosis and therapy of ketosis in non-gravid and non-lactating guinea pigs

by Nicole S. Schmid[1], Dr med vet; Marcus Clauss[1], Prof Dr med vet; Udo Hetzel[2], Dr med vet; Barbara Riond[3], Dr med vet; Monika Bochmann[1,4], Dr med vet; Jean-Michel Hatt[1], Prof Dr med vet

Content:

1. Detailed treatment plan of one animal during the feeding period

2. Supplement to table 1: basic data used for statistics

3. SOP: Liver Histology scoring

4. Electron microscopy images of the liver

1. Detailed treatment plan of one animal during the feeding period

During the feeding period, one slim male animal had an ocular injury (perforated infected corneal ulcer) and had to be treated according to the ophthalmologists' instructions for 14 days. It received Serum-eye drops and Vigamox® [a] (Moxifloxacin) eye drops each 3 to 4 times a day, 0.6ml Metacam® [b] for dogs (Meloxicam, 1mg/kg) and 0.56ml Baytril® [c] 2.5% (Enrofloxacin, 15mg/kg) each per oral once a day and daily probiotic support (Benebac® [c]). This animal later occurred as an outlier in the bile acid measurements and was excluded from statistical evaluation.

[a] Novartis Pharma AG, 6343 Rotkreuz, Switzerland
[b] Boehringer Ingelheim GmbH, 4002 Basel, Switzerland
[c] Provet AG, 3421 Lyssach, Switzerland

Supplement to Table 1.

Basic data used for statistics of table 1.

		animals without therapy				animals with therapy			
		female		male		female		male	
		slim	obese	slim	obese	slim	obese	slim	obese
Amount of animals (n)		4	4	3	3	3	3	3	4
Lipidosis score	**median**	**1.5**	**1**	**0.5**	**0.75**	**0.75**	**0.5**	**1.25**	**0.75**
	mean	1.75	1.188	0.5	0.75	0.917	0.333	1.25	1.188
	SD	1.323	0.774	0.5	0.25	0.52	0.289	0.75	1.477
	1st quartile	0.875	0.688	0.25	0.625	0.625	0.25	0.875	0.188
	3rd quartile	2.375	1.5	0.75	0.875	1.125	0.5	1.625	1.75
	min	0.5	0.5	0	0.5	0.5	0	0.5	0
	max	3.5	2.25	1	1	1.5	0.5	2	3.25
Glycogen score	**median**	**1.375**	**4.125**	**1.25**	**4.75**	**1.5**	**6.25**	**2.25**	**7.5**
	mean	1.5	4	1.5	4.83	1.5	5.83	2.25	7.13
	SD	0.979	0.612	0.661	0.629	1.25	1.422	0.354	0.968
	1st quartile	0.875	3.625	1.125	4.375	0.875	5.25	2	6.875
	3rd quartile	2	4.5	1.75	5	2.125	6.625	2.5	7.813
	min	0.5	3.25	1	4.25	0.25	4.25	1.75	5.75
	max	2.75	4.5	2.25	5.5	2.75	7	2.75	8
Degeneration score	**median**	**0**	**2.125**	**0**	**1.75**	**0**	**3.5**	**0**	**2.5**
	mean	0.063	2	0.083	1.667	0.083	3.417	0	2.938
	SD	0.125	0.54	0.144	0.629	0.144	0.144	0	1.491
	1st quartile	0	1.813	0	1.375	0	3.375	0	2.188
	3rd quartile	0.063	2.313	0.125	2	0.125	3.5	0	3.375
	min	0	1.25	0	1	0	3.25	0	2
	max	0.25	2.5	0.25	2.25	0.25	3.5	0	4.75
Total liver damage score	**median**	**1.5**	**3.25**	**0.5**	**2.5**	**0.75**	**3.75**	**1.25**	**4**
	mean	1.813	3.188	0.583	2.417	1	3.75	1.25	4.125
	SD	1.248	0.315	0.629	0.382	0.661	0.25	0.75	1.561
	1st quartile	0.938	3.125	0.25	2.25	0.625	3.625	0.875	3.063
	3rd quartile	2.375	3.313	0.875	2.625	1.25	3.875	1.625	5.063
	min	0.75	2.75	0	2	0.5	3.5	0.5	2.5
	max	3.5	3.5	1.25	2.75	1.75	4	2	6
Bile acids	median	20.3	34.55	6.9	37.2	12.4	41.7	74.75	71.6
	**mean**	**21.925**	**36.625**	**6.367**	**35.1**	**16.333**	**49.400**	**74.750**	**70.225**
	SD	12.541	13.249	2.542	5.274	12.763	18.494	8.132	40.703
	1st quartile	14.45	29.95	5.25	33.15	9.2	38.85	71.875	38.025
	3rd quartile	27.775	41.225	7.75	28.1	21.5	56.1	77.625	103.8
	min	8.9	22.9	3.6	29.1	6	36	69	29.7
	max	38.2	54.5	8.6	39	30.6	70.5	80.5	108
ALT	median	41	29.5	41	34	42	39	51	55
	**mean**	**40.75**	**41.5**	**45.667**	**34**	**43.667**	**41**	**51**	**56**
	SD	3.304	28.641	8.083	5	5.686	9.165	1.414	11.015
	1st quartile	38.5	25.25	41	31.5	40.5	36	50.5	48
	3rd quartile	43.25	45.75	48	36.5	46	45	51.5	63
	min	37	23	41	29	39	33	50	45
	max	44	84	55	39	50	51	52	69

3. SOP: Liver Histology Scoring

The following pictures of histology slides were taken from a representative location at a magnification of x200.

A score from 0 to 3 was given for each of the three regions and a total score assessed.

Legend:

Severity: 0 = None, 1 = slight (<30%), 2 = moderate (30-70%), 3 = severe (>70%)

Location: I = periportal, II = intermediate, III = centrilobular region

Scores are written as a total score of the periportal, intermediate and the centrilobular score

Degeneration score:

Values from 0-3 for each location I-III

**F17-0390**	**F17-0384**
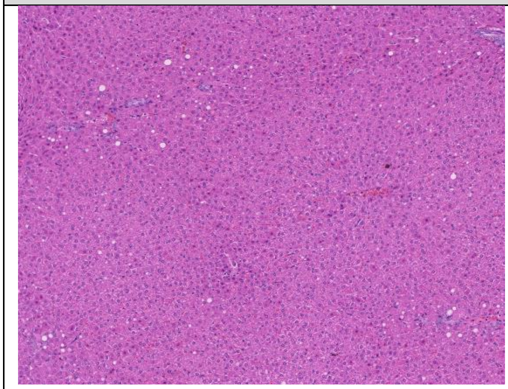	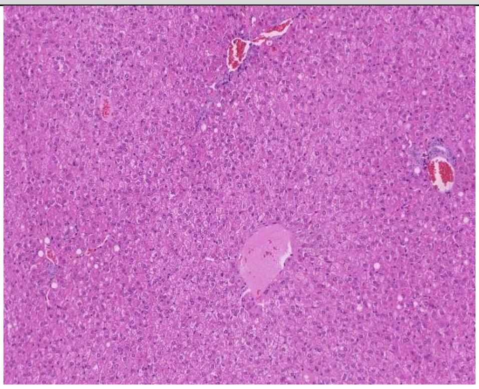
Total severity score: 0<br>(periportal: 0/intermediate: 0/centrilobular: 0)	Total severity score: 2<br>(periportal: 0/intermediate: 1/centrilobular: 1)
**F17-0392**	**F17-0398**
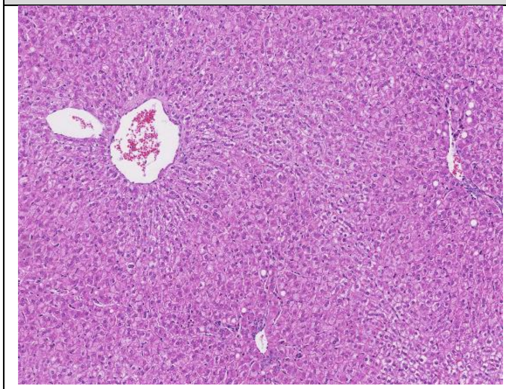	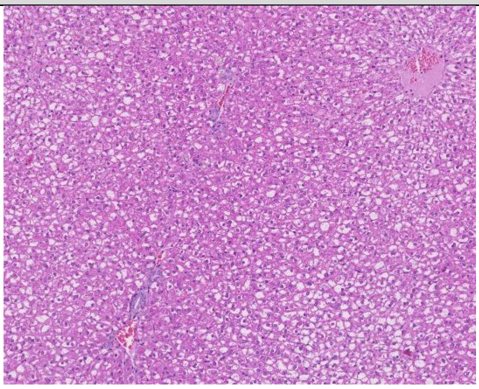
Total severity score: 3.5<br>(periportal: 0.5/intermediate: 1/centrilobular: 2)	Total severity score: 5.5<br>(periportal: 1/intermediate: 2/centrilobular: 2.5)

Lipidosis score:

Values from 0-3 for each location I-III

**F17-0374**	**F17-0376**
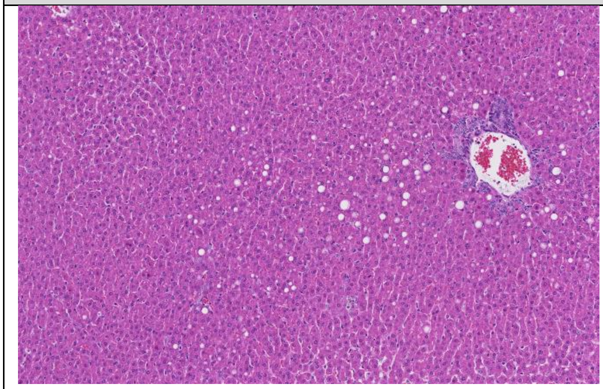	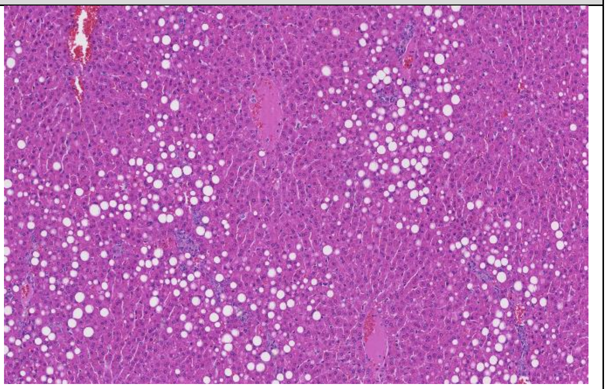
Total severity score: 1<br>(periportal: 1/intermediate: 0/centrilobular: 0)	Total severity score: 3<br>(periportal: 2/intermediate: 1/centrilobular: 0)
**S17-1105***	**S17-1238***
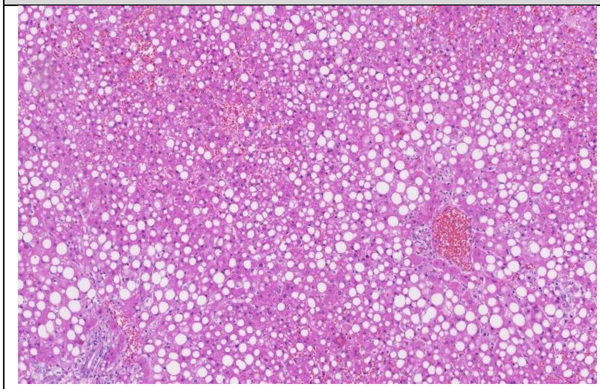	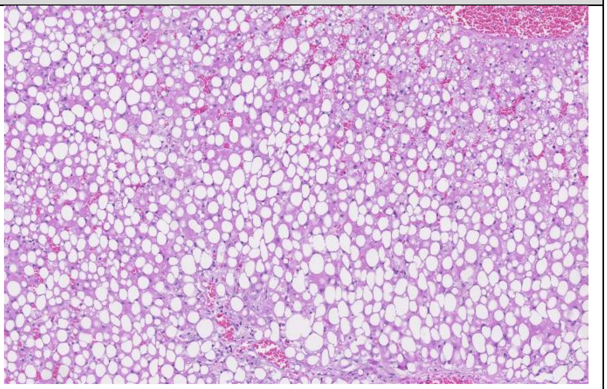
Total severity score: 7<br>(periportal: 3/intermediate: 2/centrilobular: 2)	Total severity score: 9<br>(periportal: 3/intermediate: 3/centrilobular: 3)

* S17-1105 and S17-1238 were guinea pigs that died at the clinic and had anamnesis of anorexia. The pictures were taken to asses the histology score, as our animals did not show as severe lipidosis.

Glycogen content score:

Values from 0-3 for each location I-III

**F17-0388**	**F17-0382**
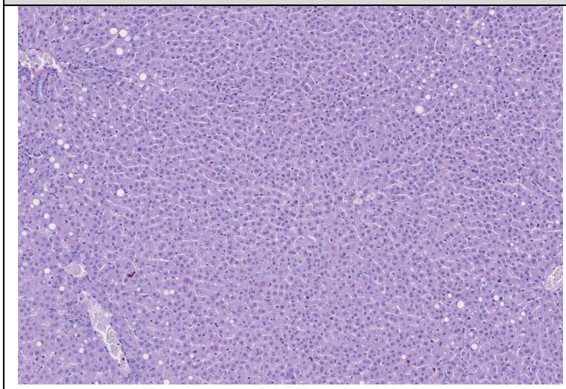	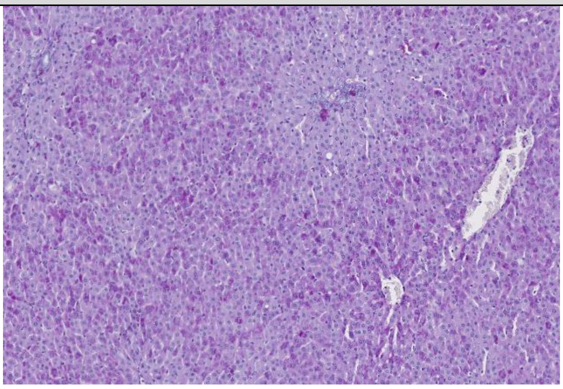
Total severity score: 0.5<br>(periportal: 0/intermediate: 0.5/centrilobular: 0)	Total severity score: 2.5<br>(periportal: 0.5/intermediate: 1/centrilobular: 1)
**F17-0384**	**F17-0397**
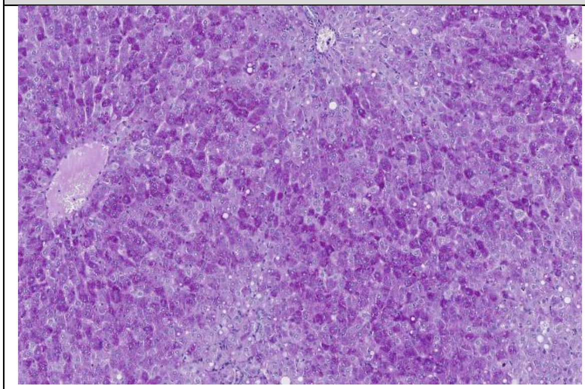	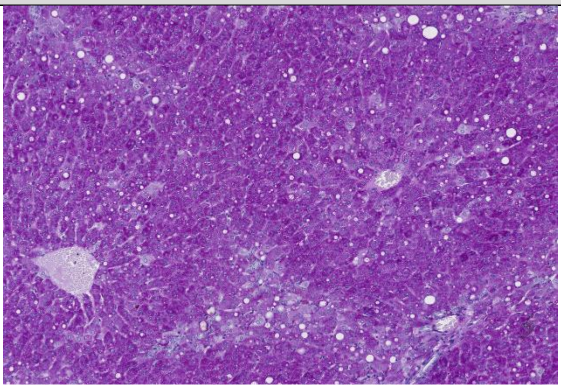
Total severity score: 4.5<br>(periportal: 0.5/intermediate: 2/centrilobular: 2)	Total severity score: 7.5<br>(periportal: 2/intermediate: 3/centrilobular: 2.5)

4. Electron microscopy images

A)

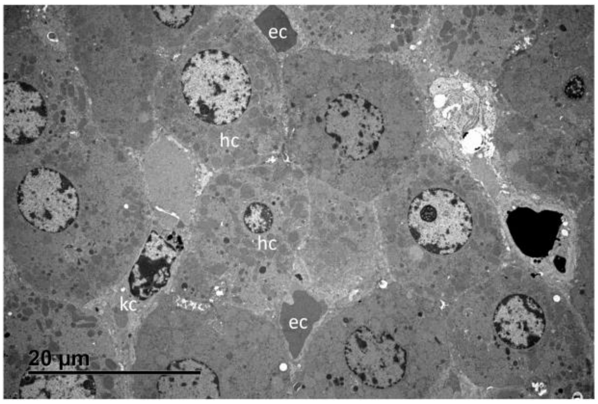

B)

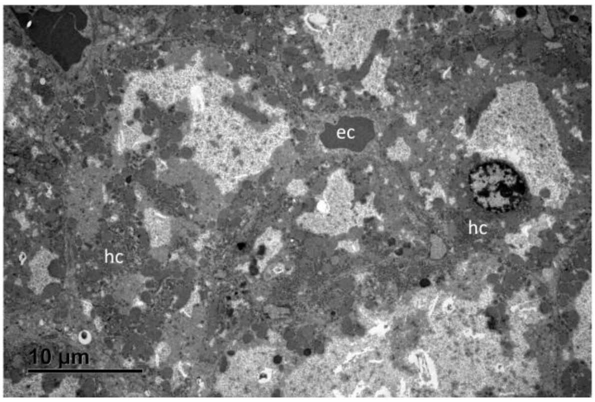

ec = sinusoidal erythrocytes, hc = hepatocyte, kc = Kupffer cells

A) Ultrathin section of normal guinea pig liver (group: slim female without therapy) with regular hepatocytes (hc) with round hypochromatic nuclei, moderately electron dense, mitochondria rich cytoplasm, few sinusoidal erythrocytes (ec) and Kupffer cells (kc).

B) Section of altered guinea pig liver (group: obese male with therapy) with irregularly shaped hepatocytes (hc) with indistinct cell borders, partly electron lucent disintegrated cytoplasm, mitochondrial condensation and aggregation (asteriscs), and electron dense nuclear chromatin (nc).
